# Novel and classical renal biomarkers as evidence for the nephroprotective effect of *Carica papaya* leaf extract

**DOI:** 10.1042/BSR20181187

**Published:** 2018-09-21

**Authors:** Ibtsam Gheith, Abubakr El-Mahmoudy

**Affiliations:** 1 Department of Clinical Laboratory Sciences, Faculty of Applied Medical Sciences, Taibah University, Medinah 344, Kingdom of Saudi Arabia; 2 Department of Biotechnology, Animal Health Research Institute, Dokki 11843, Egypt; 3 Department of Pharmacology, Benha University Faculty of Veterinary Medicine, Moshtohor 13736, Egypt

**Keywords:** biomarkers, clinical pathology, medicinal plants, phytomedicine, pharmacology

## Abstract

The present study is aimed at utilization of novel and classical kidney function biomarkers to evaluate the nephroprotective potential of *Carica papaya* leaf extract (CPLE) in gentamicin nephrotoxicity model in albino rats. The used classical biomarkers were urea and creatinine; while the new biomarkers were Kidney injury molecule-1 (KIM-1) and Clusterin. Forty-five male albino rats were assigned into five groups and subjected to different treatments for nine consecutive days (vehicles; gentamicin, 100 mg/kg, subcutaneously; ascorbic acid, 200 mg/kg, orally; CPLE, 150 and 300 mg/kg b wt., orally). Three rats/group were killed on days 3, 6, and 9 for blood and tissue samples for renal and oxidation markers. Gentamicin resulted in significant increase in urea and creatinine only by the end of the experimental course; while the novel biomarkers were evident as early as 3 days upon gentamicin injection. When concurrently administered with gentamicin, CPLE significantly protected kidney tissues against gentamicin nephrotoxic effects indicated by decrement of both the novel and the classical standard biomarkers, in a dose-dependent manner. CPLE-mediated protection was attributed to its antioxidant potential indicated by significant inhibition of malondialdehyde (MDA) levels in both serum and kidney homogenates. The results were further supported by histopathological examination that revealed considerable amelioration of the pathological microscopic alterations induced by repeated gentamicin injection. Phytochemical analysis of CPLE indicated presence of tannins and flavonoids. These data may suggest CPLE, based on improvement of both classical and novel renal markers, as a highly potent nephroprotective and antioxidant from natural source that could be a good remedy in conditions associated with renal disorders.

## Introduction

Kidney is a major multifunctional organ in the body. Briefly, it is responsible for regulation of fluid and acid-base balances; clearance of toxins whether endogenous (as urea and creatinine) or exogenous (as drugs); re-absorption of some beneficial molecules as glucose and amino acids; regulation of blood pressure; production of some hormones, as erythropoietin; and activation of calciferol. Amongst these functions, clearing xenobiotics may subject the kidney to acute or chronic injury according to the degree and time of exposure. The clinicochemical standard parameters applied for the evaluation of renal function are serum creatinine and blood urea nitrogen. These markers have been described since 1904 [1] and 1952 [2], respectively; and are still the routine standards in the minimal diagnosis of the kidney ability. However, the sensitivity of urea and serum creatinine is considered, recently, as poor if it is taken in mind that the kidney is capable of compensating the renal mass loss by hyperfunctioning of the still-healthy mass, and the reduction in renal functionality occurs only after loss of approximately two-thirds of renal biomass [3]. Moreover, these two parameters can easily be influenced by many nutritional, physiological, and extra-renal pathological factors. Therefore, a scientific need to discover newer early-stage sensitive and specific renal biomarkers has emerged. Collaborative efforts done by specialists [4] as well as others have resulted in introducing several urinary biomarkers that have been approved by European Medicine Agency (EMA) and Food and Drug Administration (FDA), for early and specific detection of nephrotoxicity in rats. These biomarkers include, β_2_-microglobulin, Cystatin C, kidney injury molecule-1 (KIM-1), Clusterin, N-acetyl-β-d-glucosaminidase (NAG), Osteopontin, tissue inhibitor of matrix metalloproteinase-1 (TIMP-1), Glutathione-S-transferase (GST)-α, Neutrophil gelatinase associated lipocalin (NGAL). These new markers were judged by the regulatory authorities (EMA and FDA) to be acceptable for detection of acute drug-induced renal toxicity and to provide complementary information to the already used standard parameters. Some of the above-mentioned novel renal markers, in addition to the standard conventional markers (urea and creatinine) were applied in the present study to assess the possible nephroprotective role of aqueous extract of a plant with interesting nutraceutical properties, that is *Carica papaya*.

*C. papaya* (also known as pawpaw or papaya) is amongst the most important nutritional, pharmaceutical, and economic plants distributed worldwide. It is a leguminous tree with fruits belonging to *Caricacae* family, Plantae Kingdom [5]. *Papaya* tree grows up to approximately 9 m (height). It has hollow, herbaceous stem, that is usually unbranched. The leaves are deeply lobed, borne on long, hollow petioles emerging from the stem apex. Fruits have smooth skin, vary widely in size and shape, contain many seeds surrounded by a smooth yellow to orange-red flesh with light sweet taste [6]. The plant has been used in traditional medicines and some of its pharmacological and health potentials have been proved. Different extracts from different parts of the plant were reported to exhibit wound healing [7], antiulcer [8], anti-inflammatory [9], antihyperlipidemic [10], antifungal [11], antimalarial and for dengue fever [12,13], immunostimulant [14], thrombocytotic [15,16], anti-nociceptive [17], anticancer [18], antipyretic [19], digestive [20], and antidiabetic [21] activities.

Oxidative stress is a predisposing factor and direct cause of various disorders in both man and animals, including renal ones. This fact encouraged pharmacologists to utilize antioxidants, especially those of natural source, for prophylaxis and treatment of such disorders and improving the health status of normal subjects as well [22].

Based on the above-mentioned background, the present study, therefore, was adopted to utilize some of the novel renal markers, namely, KIM-1 and Clusterin, along with the classical ones (urea and creatinine) to evaluate the renal function in gentamicin-nephrotoxicity model with and without challenge by the aqueous extract of *C. papaya* leaves. The underlying mechanisms and active principles have been also tried.

## Materials and methods

### Plant part used

The fresh leaves of *C. papaya* were collected from our local environment and identified by a Botany specialist.

### Extraction procedure

The adopted methodological procedures of extraction were modified after [23]. The method that keeps most constituents and simulates, to a large extent, was the traditional method used for the plant leaves in folk medicine. Plant leaves were refluxed in running tap water and then with bi-distilled water, shade dried at room temperature and chopped using clean knife and flat board. The extract was prepared by macerating a weighed amount (200 g) of the small parts of the leaves in a known volume (1.5 l) of aqueous: organic solvent (bi-distilled water:ethanol, 70:30, *v/v*) in covered Erlenmeyer flasks. Maceration continued for 48 h under refrigeration with occasional shaking. The hydroethanolic extract was filtered and then concentrated using a gently shaking water bath at 56°C in clean, pre-weighed, labeled glass beakers. The weight of the obtained semisolid residue (yield) was calculated and re-constituted in a measured amount of isotonic saline (NaCl 0.85%, *w/v*). The reconstituted extract concentration was adjusted at 15 and 30 mg/ml in isosaline; where a rat weighing 200 g receives 2 ml of the corresponding extract to be equivalent for the doses 150 and 300 mg/kg, respectively. Extracts for phytochemical testing were prepared appropriately according to the test applied as mentioned later.

$$\text{Yield }\%\text{ was calculated as}:\frac{Extracted\;residue\;weight}{Original\;seed\;weight}\times 100$$

### Chemicals

Gentamicin was obtained as the patent preparation Garamycin® (manufactured by: Memphis Co. for Pharm. & Chem. Ind. (MEMCO), Egypt; under authority of: Schering-Plough Corporation, U.S.A.; #050039) that is formulated as ampoules of 1 ml containing 40 mg gentamicin as sulphate. Ascorbic acid was obtained as Vitacid-C® (CID, Haram, Egypt) that is formulated as effervescent tablets of 1000 mg. It was solubilized in water and thoroughly shaken to get a concentration of 40 mg/ml. Thiobarbituric acid was purchased from Sigma–Aldrich (St. Louis, United States). All other chemicals were of analytical grade and available from local distributers.

### Study design

Forty-five, male, weighing approximately 200 g albino rats, 6 weeks of age were humanely used in the present study. The rats were kept hygienically in conditioned room with free access to clean water and balanced diet. After acclimatization, rats were randomly separated into five groups (nine in each) in separate suitably sized cages and subjected to different treatments. Control group (C) was injected and orally administered only with the vehicles (sterile water and isosaline) of gentamicin and *C. papaya* leaf extract (CPLE), respectively, at the corresponding time points of other treated groups; Diseased group (D) was injected with gentamicin (100 mg/kg, intraperitoneally, for nine successive days, and orally administered with only the vehicle of CPLE at the corresponding time points; Standard group (S) was injected with gentamicin in the same manner as group-D, and orally administered ascorbic acid in isosaline (200 mg/kg, daily for 9 days, using stomach tube) as standard antioxidant; and treated groups (CPLE-SD and CPLE-LD) injected with gentamicin in the same manner as of group-D, and orally administered with CPLE in isosaline (150 and 300 mg/kg, respectively, daily for 9 days, using stomach tube) as tested nephroprotective. Doses of CPLE were average doses from the dosage range reported in literatures (500–1000 mg, three times daily for man), converted into those of rats using conversion factors after Paget and Barnes [24]. Along the course of the experimental period, three rats/group were killed on days 3, 6, and 9. Before killing, blood samples were taken from the medial canthus venous plexus under light ether anesthesia. Each blood sample was received into a plain sampling tube, left to coagulate, centrifuged, and serum was collected in labeled Eppendorf tubes and used for determination of urea, creatinine, KIM-1, Clusterin and malondialdehyde (MDA). After killing, both kidneys were picked out; one into Falcon tube containing ice-cold isosaline to be homogenated for determination of tissue MDA; while the second kidney was received into a bottle containing 10% formol-saline for histopathology. Urine samples were taken in the same sampling points via urinary bladder puncture for determination of urinary urea, creatinine, KIM-1 and Clusterin. All procedures were ethical to animals and performed with merciful and humane manner under light ether anesthesia and adhered to principles published by International Council for Laboratory Animal Science (ICLAS).

### The classical renal biomarkers assays

Estimating serum and urine classical renal biomarkers was carried out spectrophotometrically (Jenway®, Model 6500, Germany) using commercial diagnostic kits purchased from Analyticon® Biotechnologies AG (Lichtenfels, Germany) following the instructions of the manufacturer. The principles of estimation procedure of urea and creatinine were derived from Krieg et al. [25] and Slot [26], respectively.

### The novel renal biomarkers assays

Two novel renal function biomarkers have been assessed, namely KIM-1 (a transmembrane protein expressed by tubule epithelial cells in response to injury) and Clusterin (a secreted glycoprotein involved in cell adhesion and apoptosis in some tissues including proximal and distal renal tubules) in serum and urine of experimental rats. The markers have been estimated by ELISA using microplate reader (Spectrostar®, BMG-LabTech GmbH, Ortenberg, Germany) and commercial diagnostic kits obtained from BioCat® GmbH, Heidelberg, Germany according to instructions of the manufacturer. After recording absorbance from the provided standard and all samples at 450 nm, the concentrations of the markers have been calculated after considering dilution factors of serum and urine samples.

### The antioxidant assays

MDA concentration in serum and kidney homogenate was measured as an indicator for the extent of membrane lipid peroxidation, following the original method of Ohkawa et al. [27] with minor modifications. This was performed using thiobarbituric acid solution, which reacts with MDA giving pinkish color that could be measured spectrophotometrically. First, kidney homogenate was prepared by using an electric tissue homogenizer, while keeping container tubes in ice box. One gram of the tested kidney tissue was immersed in 9 ml of 100 mmol KCl buffer (cold, pH 7.4, containing EDTA 0.3 mM), i.e. 1:10 w/v (10%), and thoroughly homogenized. The homogenate was then centrifuged at 5000 rpm for 20 min at 4°C. The supernatant was used for the assay. The reaction mixture consisted of 0.2 ml of 8.1% SDS, 1.5 ml of 20% acetic acid solution (adjusted at pH 3.5 with NaOH) and 1.5 ml of 0.8% aqueous solution of thiobarbituric acid was added to 0.2 ml of the prepared kidney homogenate. The mixture was completed to 4.0 ml with distilled water and heated at 95°C for 60 min. After cooling under tap water, 1.0 ml distilled water and 5.0 ml of the mixture of n-butanol and pyridine (15:1 v/v) was added and the mixture was vigorously shaken and recentrifuged. The organic layer (upper one) was taken out and its absorbance was measured at 532 nm. The level of MDA in each sample was calculated from absorbance values compared with that of 1,1,3,3-tetramethylpropane (TMP; 20 nM) as external standard for MDA; and expressed as nM/g wet wt.

### Histopathological examination

A kidney from each experimental rat was picked out and immediately placed in 10% formalin solution; processed for sectioning, staining, and microscopic examination as described by Bancroft and Gamble [28]. Samples were allowed to fix for a 24-h period. The fixed samples were then gently washed under slowly running water overnight. The clean fixed samples were then allowed to dehydrate in a series of increased concentrations of ethanol starting in 70% and finalizing in absolute alcohol. The fixed, clean, dehydrated samples were placed in xylol for 3 h to clear, and then placed in melted paraffin wax tissue boxes. The wax containing tissue specimens was left to solidify and then sections of 4–6 µm thickness were obtained using a rotary microtome. Staining procedure started with removal of wax from the microsections by two changes in ethanol (absolute; 5 min each). Ethanol was washed away with water. Sections were stained with H&E for 10 min, and then the extra stains were gently flushed under running water for 15 min. The stained microsections were dehydrated by alcohol series as mentioned above, then allowed to clear in xylol and finally covered with DBX. The prepared microsection slides were examined microscopically and interpreted by a specialist.

### Phytochemical analysis

Phytochemical detection tests for presence of phenols and flavonoids in leaf extracts of *C. papaya* were carried out as described previously [29]. The tests were performed as triplicates and given marks from (−) to (+++) according to the strength of the color or precipitate that appeared. Presence of tannins and/or other phenolic compounds in the leaf extract was determined using gelatin, lead acetate, phenazone, ferric chloride, hydrochloric acid, and vanillin tests; while presence of flavonoids was determined by Shinoda’s, Wislon’s, and alkaline reagent tests.

### LC-MS analysis

The used chromatographic system composed of an Agilent® 1100 series (Agilent Technologies Inc., CA, U.S.A.) including binary pump (G1312B), equipped with on-line solvent degasser (G1379B), Auto-sampler (G1367E), thermo-stated column (G1316A) and temperature module (Palo Alto, CA, U.S.A.). The chromatographic system was interfaced with an Applied Biosystems 4000 QTRAP® MS system with Turbo V™ ion source. The whole system was controlled using Analyst® software version 1.4.1. Leaves extract was subjected to analysis using a mobile phase of water:acetonitrile (80:20 v/v) with a column temperature of 25°C and a flow rate of 1.5 ml/min. The mode of ESI was applied and mass/charge (m/z) range of 50–1000. Analyzed phytochemicals were identified by the aid of comparing the mass spectral data with Wiley 275 mass spectral library.

### Data presentation and analysis

Data are expressed as mean ± S.E.M. of three separate observations at each sampling point. Observations were compared at a particular time point using ANOVA followed by LSD as post-hoc test at a *P*-level of 0.05. All procedure of statistics and graphing were done using the computer program GraphPad Prism® version 6 (GraphPad Inc., CA, U.S.A.).

### Ethics approval and consent to participate

All procedures were ethical to animals and performed with merciful and humane manner under light ether anesthesia and adhered to principles published by ICLAS (reference number: not applicable).

## Results

The yield % of the shade-dried *C. papaya* leaves when macerated in hydroethanol (70:30, v/v) and evaporated was high, equaled 32.4%. The extract was gummy in consistency and of green color.

As shown in Table 1, *CPLE* impeded the elevation of classical renal markers, urea and creatinine, in serum and urine of gentamicin-treated rats; the impedance was significant compared with the values of gentamicin only treated rats; and was comparable with that exhibited by the standard drug, ascorbic acid. These results were observed only on the last sampling point (day 9). Although observed on the sixth day yet were non-significant because of modest elevation of urea and creatinine.

**Table 1:** Classical renal biomarkers in serum and urine after intraperitoneal injection of gentamicin (100 mg/kg b wt., for nine consecutive days) and oral administration of CPLE (150 and 300 mg/kg b wt., for nine consecutive days) to rats compared with those after the standard ascorbic acid (200 mg/kg b wt., orally, for nine consecutive days) and normal control (Saline); (mean ± S.E.M.; *n*=3 at each time point)

Group	Days	Serum		Urine	
		Urea (mg/dl)	Cr. (mg/dl)	Urea (g/dl)	Cr. (mg/dl)
C	3	32.47 ± 1.73	0.55 ± 0.03	12.56 ± 0.32	84.67 ± 2.06
	6	32.69 ± 1.45	0.59 ± 0.02	12.67 ± 0.35	85.11 ± 3.01
	9	33.17 ± 2.02	0.61 ± 0.03	12.80 ± 0.37	86.50 ± 2.63
D	3	33.43 ± 1.47	0.65 ± 0.03	13.01 ± 0.18	90.33 ± 2.62
	6	48.23 ± 1.75^1^	1.05 ± 0.09^1^	15.17 ± 0.45	98.03 ± 3.73^1^
	9	122.70 ± 4.35^1^	3.57 ± 0.25^1^	40.33 ± 1.49^1^	156.47 ± 3.59^1^
S	3	30.33 ± 2.02	0.61 ± 0.02	12.61 ± 0.17	86.01 ± 2.39
	6	35.03 ± 2.34	0.70 ± 0.02	13.43 ± 0.30	89.67 ± 2.68
	9	49.33 ± 2.39^2^	1.01 ± 0.07^2^	20.83 ± 0.84^2^	101.33 ± 3.18^2^
TSD	3	32.67 ± 1.45	0.63 ± 0.02	13.11 ± 0.15	87.66± 1.46
	6	39.17 ± 1.56	0.87 ± 0.05	14.43 ± 0.26	95.16 ± 2.48
	9	70.13 ± 2.40^2^	2.03 ± 0.15^2^	26.17 ± 0.98^2^	111.33 ± 2.83^2^
TLD	3	30.34 ± 2.31	0.64 ± 0.02	12.81 ± 0.14	85.67 ± 1.23
	6	34.57 ± 1.56	0.82 ± 0.06	14.13 ± 0.26	93.16 ± 2.39
	9	59.13 ± 3.18^2^	1.13 ± 0.09^2^	21.37 ± 0.85^2^	103.67 ± 2.82^2^

Abbreviations: TLD, treated with larger dose of CPLE; TSD, treated with small dose of CPLE.

^1,2^ mean significantly (*P*<0.05) different from Normal and Diseased, respectively, on the corresponding day.

Unlike the classical markers’ data, Table 2 shows that the novel markers, KIM-1 and Clusterin, were elevated in gentamicin group starting from the third day samples. The extract again guarded against increase in such markers.

**Table 2:** Novel renal biomarkers in serum and urine after intraperitoneal injection of gentamicin (100 mg/kg b wt., for nine consecutive days) and oral administration of CPLE (150 and 300 mg/kg b wt., for nine consecutive days) to rats compared with those after the standard ascorbic acid (200 mg/kg b wt., orally, for nine consecutive days) and normal control (Saline); (mean ± S.E.M.; *n*=3 at each time point)

Group	Days	Serum		Urine	
		KIM-1 (ng/ml)	Clu. (ng/ml)	KIM-1 (ng/ml)	Clu. (ng/ml)
C	3	0.04 ± 0.009	160.67 ± 6.36	0.47 ± 0.02	20.63 ± 2.36
	6	0.05 ± 0.001	174.13 ± 5.52	0.51 ± 0.03	24.11 ± 1.71
	9	0.05 ± 0.006	179.51 ± 2.68	0.60 ± 0.02	25.50 ± 2.43
D	3	212.73 ± 5.67^1^	8983.3 ± 101.4^1^	29.66 ± 1.22^1^	598.53 ± 7.57^1^
	6	198.43 ± 3.82^1^	8483.4 ± 88.2^1^	26.33 ± 1.28^1^	698.13 ± 8.71^1^
	9	151.33 ± 4.62^1^	8220.1 ± 74.6^1^	22.34 ± 1.45^1^	798.67 ± 6.96^1^
S	3	98.33 ± 2.18^2^	3003.8 ± 23.1^2^	7.53 ± 0.73^2^	101.01 ± 4.9^2^
	6	80.73 ± 2.84^2^	2215.3 ± 25.2^2^	5.81 ± 0.36^2^	149.4 ± 4.63^2^
	9	49.13 ± 2.39^2^	971.7 ± 16.4^2^	3.93 ± 0.23^2^	168.6 ± 5.81^2^
TSD	3	118.3 ± 2.71^2^	3993.7 ± 26.5^2^	14.67 ± 0.88^2^	300.7 ± 5.21^2^
	6	99.33 ± 2.96^2^	3505.5 ± 24.6^2^	11.83 ± 0.73	350.6 ± 6.36^2^
	9	70.10 ± 2.40^2^	2783.6 ± 29.3^2^	8.87 ± 0.75^2^	378.7 ± 5.82^2^
TLD	3	109.30 ± 3.81^2^	3196.7 ± 11.7^2^	10.73 ± 0.48^2^	130.43 ± 3.76^2^
	6	88.33 ± 2.06^2^	2373.3 ± 31.8^2^	8.83 ± 0.36^2^	169.36 ± 4.63^2^
	9	59.67 ± 3.18^2^	996.6 ± 8.82^2^	6.96 ± 0.43^2^	192.33 ± 4.81^2^

Abbreviations: TLD, treated with larger dose of CPLE; TSD, treated with small dose of CPLE.

^1,2^ mean significantly (*P*<0.05) different from Normal and Diseased, respectively, on the corresponding day.

*CPLE* exhibited potent antioxidant activity indicated by decrement of MDA production in the kidney tissue upon giving gentamicin (Table 3).

**Table 3:** MDA as lipid peroxidation biomarker in serum (nmol/ml) and kidney homogenate (nmol/g wet weight of kidney tissue) after intraperitoneal injection of gentamicin (100 mg/kg b wt., for nine consecutive days) and oral administration of CPLE (150 and 300 mg/kg b wt., for nine consecutive days) to rats compared with those after the standard ascorbic acid (200 mg/kg b wt., orally, for nine consecutive days) and normal control (Saline); (mean ± S.E.M.; *n*=3 at each time point)

Group	Days	MDA	
		Serum (nmol/ml)	Kidney hom. (nmol/g wet wt)
C	3	1.93 ± 0.12	5.17 ± 0.26
	6	2.20 ± 0.13	5.46 ± 0.23
	9	2.53 ± 0.16	5.91 ± 0.19
D	3	10.43 ± 0.82^1^	18.13 ± 1.24^1^
	6	12.13 ± 0.85^1^	31.35 ± 1.93^1^
	9	15.76 ± 1.45^1^	34.77 ± 1.65^1^
S	3	4.07 ± 0.15^2^	5.82 ± 0.18^2^
	6	6.73 ± 0.14^2^	9.96 ± 0.72^2^
	9	6.13 ± 0.26^2^	13.70 ± 0.73^2^
TSD	3	6.06 ± 0.17^2^	5.83 ± 0.18^2^
	6	9.96 ± 0.49^2^	38.53 ± 2.61^2^
	9	11.13 ± 0.64^2^	10.56 ± 0.31^2^
TLD	3	5.60 ± 0.23^2^	7.86 ± 0.41^2^
	6	7.03 ± 0.20^2^	11.90 ± 0.78^2^
	9	7.06 ± 0.17^2^	14.83 ± 0.74^2^

Abbreviations: TLD, treated with larger dose of CPLE; TSD, treated with small dose of CPLE.

^1,2^ mean significantly (*P*<0.05) different from Normal and Diseased, respectively, on the corresponding day.

**Figure 1:**
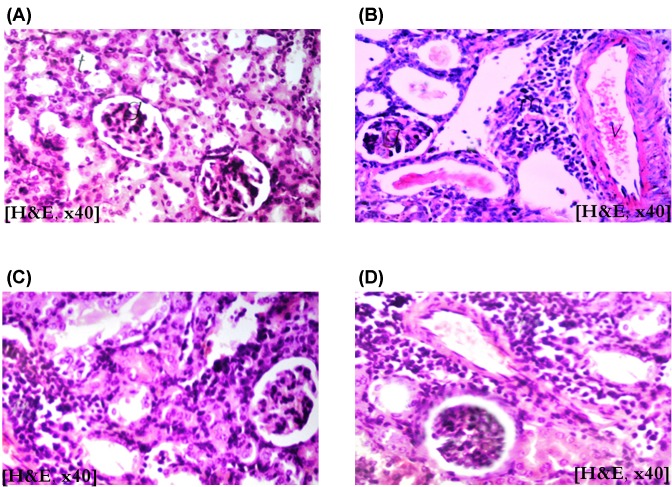
Histopathological examination Photographs for the kidney sections of control (**A**), gentamicin-treated (**B**), gentamicin-treated with concurrent ascorbate administration (**C**), and gentamicin-treated with concurrent administration of 300 mg/kg CPLE (**D**) (H&E ×40).

Histopathological findings came supportive to and parallel to the clinicochemical ones. Repeated gentamicin injection resulted in appearance of focal inflammatory cellular infiltration in between the tubules and surrounding blood vessels that are congested, associated with appearance of homogeneous eosinophilic casts in the lumen of cystically dilated tubules. These gentamicin-induced degenerative changes in the kidney tissue were ameliorated, to a great extent, upon concurrent administration of CPLE; as well as of the standard ascorbic acid; the pathological changes were limited to focal inflammatory cellular infiltration in between the glomeruli and tubules at the cortex (Figure 1).

Phytochemical analysis revealed presence of tannin- and flavonoid compounds in the examined extract (Table 4). While LC-MS analysis revealed 21 phytochemical compounds with spectral masses as follows: tocopherol (430.72), ascorbic acid (176.13), carpaine (466.71), deoxykaempferol (270.25), kaempferol (286.24), deoxyquercetin (286.25), quercetin (302.24), dicoumarol (336.31), coumaroylquinic acid (338.32), coumarin (146.15), folic acid (441.41), cystine (121.16), homocysteine (135.19), cysteine sulphoxide (121.16), l-glutamic acid (147.13), *p*-coumaroyl alcohol (150.18), dimethoxy phenol (154.17), umbelliferone (162.15), phenylalanine (165.19), caffeoyl alcohol (166.18), and methyl nonyl ketone (170.30).

**Table 4:** Qualitative phytochemical analysis of the extract of *C. papaya* leaves

Active principle group	Test	Result
Tannin	Gelatin	++
	Lead acetate	++
	Phenazone	++
	FeCl_3_ test	++
Phlobatannin	Hydrochloric acid test	++
Gallic acid	Vanilin test	++
Flavonoids	Shinoda’s test	+++
	Wilson’s	+++
	Lead acetate	+++
	Alkaline reagent	+++

+ denotes strength of positive reaction of the extract with the detecting reagent.

## Discussion

Acute kidney injury (AKI) or renal failure (ARF) is characterized by rapid, usually reversible, decline in renal function associated with rapid retention of nitrogenous waste products over a period of a few days. AKI is considered as a threatening complication following many causes, including trauma, surgical procedures, and massive administration of xenobiotics or in patients hospitalized in intensive care units [30]. ARF may be pre-renal, renal, or post-renal. Pre-renal type is a consequence of decreased renal blood flow due to hypovolemia or ischemia. The renal type occurs when there is an internal damage to the renal structures as the glomeruli, tubules, vessels, or interstitium because of exposure to xenobitics or radiation. The post-renal type occurs in case of obstruction of the urine collection structures and increase in its intra-pressure resulting in reduced GFR and renal failure [31].

ARF may be life-threatening if it is diagnosed in its later stages. Previously, laboratory diagnosis of kidney function was basically dependent on estimation of urea and creatinine. However, elevations of these two parameters occurs only after loss of approximately two-thirds of renal biomass as the healthy part is capable of compensating the lost renal mass [3]. Although still standard biomarkers, however, recently they are judged as poorly sensitive as they are elevated only in the late stages of ARF. This critical issue necessitated discovery of other biomarker for the AKI to predict and detect early renal disease. Approximately ten newer parameters have been introduced to be measured in serum and urine for early renal diagnosis, including, β_2_-microglobulin, Cystatin C, KIM-1, Clusterin, NAG, Osteopontin, TIMP-1, GST-α, NGAL [32]. These novel markers could be detected in serum and urine as early as 2–3 days of AKI; and this early elevation is considered as a highly appreciated finding for early diagnosis and protection against renal disease before its bad prognostic stage.

These biomarkers could be also applied in experimental research to evaluate the nephroprotective potentials of drugs and their nephrotoxic effects as well. In the present study, two novel biomarkers (KIM-1 and Clusterin) besides the conventional ones (urea and creatinine) have been applied to evaluate the possible nephroprotective potential of CPLE in gentamicin model of nephrotoxicity in rats. Gentamicin has been used for the treatment of severe Gram-negative infections; however, its nephrotoxicity is one of the most common causes of ARF. The clinical reports have documented that aminoglycosides levels above 2.5 μg/ml possess the major risk factors for aminoglycoside-associated nephrotoxicity [33]. The mechanism of renal failure caused by the aminoglycoside gentamicin is that it is uptaken and internalized by proximal tubular cells by binding to negatively charged phospholipids. After internalization, gentamicin is transported to lysosomes and binds to acidic phospholipid membrane, causing reduced phospholipase activity and production of lipid metabolites [34].

Additional factors underlying gentamicin nephrotoxicity include generation of superoxide anion and hydroxyl radicals, alteration of antioxidant defense systems, depletion of reduced glutathione [35]. Administration of gentamicin at a dosage of 100 mg/kg, *ip*, for more than five consecutive days is most commonly used as nephrotoxicity model in rats [30].

From the above-mentioned mechanism of gentamicin-induced nephrotoxicity, it could be hypothesized that medicinal plants that have antioxidant potentials and free radical phytochemical principles that may act as powerful nephroprotectants.

In the present study, concurrent oral administration of CPLE along with gentamicin injections resulted in significant inhibition of the elevated classical urinary biomarkers, namely urea and creatinine, both in serum and urine. This inhibition was dose-dependent, comparable with the effects of the standard agent (ascorbic acid) and evident mostly only on the last (ninth) day of the experiment. It is worthy to note that both parameters did not show significant elevations in gentamicin group in the early sampling time points (3 and 6 days). However, parallel results were observed regarding the novel markers KIM-1 and Clusterin but at all examined time points (from 3 to 9 days), where the markers showed elevations as early as 3 days in the gentamicin group, unlike the classical ones. Nephroprotective effect of CPLE demonstrated in the present study is in partial agreement with results of Nwangwa [36] who reported that *C. papaya* seed extract protected kidney in CCl_4_-model, in terms of decrement of urea, creatinine, and electrolytes except potassium. These results are also parallel to those of Madinah et al. [37] who reported that *C. papaya* seed extract protected kidney in paracetamol-induced nephrotoxicity model in terms of decrement of urea, uric acid, and creatinine.

The obtained nephroprotective results may be explained on the basis of the antioxidant properties of CPLE that are proved in the present study. The extract was found to significantly decrease the levels of MDA in serum and kidney homogenate samples compared with those recorded from gentamicin diseased control. MDA is considered as a biomarker for the extent of lipid peroxidation upon which gentamicin greatly depends on in its nephrotoxic pathogenesis as mentioned earlier. The antioxidant features of CPLE recorded in the present study may be comparable with those of Maisarah et al. [38] who studied the total antioxidant activities of different parts of *C. papaya* and stated that the antioxidant capacities of the plant were highly remarkable in the sequence of young leaves > unripe fruit > ripe fruit > seed.

The antioxidant potential of CPLE may be attributed to the tannic and flavonoid contents observed in the present study by phytochemical analysis. The present data agree with that of Juárez-Rojop et al. [39] who detected tannins and steroids and with those of Ikeyi et al. [40] who detected tannins and flavones in pawpaw leaf extracts. However, the data may be in partial disagreement with Alorkpa et al. [41] who did not detect tannins in leaves of *C. papaya* extract. The antioxidant power of CPLE could be further derived from its content of tocopherol and ascorbic acid as reported by Akhila and Vijayalakshmi [42] who conducted a phytochemical study on *C. papaya* leaf juice.

Histopathological findings observed in the present study came supportive and in line with the clinicochemical ones. Kidney samples from gentamicin group exhibited severe degenerative changes in the form of congestion, leukocytic infiltration, focal necrosis, and intra-tubular hyaline casts giving indication for acute renal damage. This finding is similar to that previously reported by Ogundipe et al. [43] who observed loss of cellular constituents of tubules, densely eosinophilic cast in the lumen of some tubules, and severe cloudy swelling and inflammation of the distal convoluted tubules. However, concurrent administration of CPLE along with gentamicin in the present study, greatly protected the kidney tissue from severe degenerative changes, where the pathological lesions were limited only to congestion and focal leukocytic infiltration giving indication to the protective effect of CPLE at the tissue level.

## Conclusion

Depending on the findings of the present study, it could be concluded that the aqueous extract of *C. papaya* leaves has a strong potential of nephroprotection based on its antioxidant activity and its content of beneficial phytochemical constituents. The leaves of *C.* papaya plant, thus, could be a good pharmaceutical source of natural nephroprotective medicines.

## Abbreviations

AKI: acute kidney injury
ARF: acute renal failure
CPLE: *Carica papaya* leaf extract
EMA: European Medicine Agency
FDA: Food and Drug Administration
GST: glutathione-S-transferase
ICLAS: International Council for Laboratory Animal Science
KIM-1: kidney injury molecule-1
MDA: malondialdehyde
NAG: N-acetyl-β-d-glucosaminidase
NGAL: neutrophil gelatinase associated lipocalin
TIMP-1: tissue inhibitor of matrix metalloproteinase-1
